# Characterisation of a novel vaccine delivery system for livestock

**DOI:** 10.1186/1476-9255-12-S1-P3

**Published:** 2015-04-16

**Authors:** Rebecca K McLean, Ann R Wood, Jayne C Hope, Gary Entrican, David J Griffiths

**Affiliations:** 1 Moredun Research Institute, Pentlands Science Park, Edinburgh, EH26 0PZ, UK; 2 Royal (Dick) School of Veterinary Science, University of Edinburgh, EH25 9RG, UK

## Background

Healthy animals perform better than those that are diseased; therefore there are significant benefits from preventing disease on farm by investigating new vaccine delivery systems. Recently, viral vectors have risen to prominence as candidates for generating immune responses. Of these viral candidates, lentiviruses are attractive as they have the ability to transduce non-dividing antigen presenting cells. For example, lentiviral vectors have been shown to produce an antigen-mediated immune response in mice, providing long term, sterile protection against Malaria[1].

## Material and methods

Transformed human embryonic kidney (293T) cells were transfected with four plasmids which encode the various vector components for the production of lentiviral vectors. Vectors produced so far contain an eGFP reporter cassette to facilitate measurement of infectivity by flow cytometry. Self-inactivating (SIN) vectors have been produced to decrease the probability of the generation of replication-competent virus[2]. To produce a SIN vector, viral enhancers and promoter sequences were deleted by Gibson Cloning leaving expression of transgenes controlled by an internal promoter. Integrase deficient lentiviral vectors were produced by introducing point mutations by PCR on the D,D-35-E motif within the integrase protein to reduce insertional oncogenesis[3].

## Results

Stable expression of antigens is important to a successful vaccine, therefore the stability of ovine lentiviral transduction was evaluated over a 4 week period with eGFP-positive cells measured periodically. 50% of cells remained eGFP-positive after 4 weeks in rapidly dividing cells suggesting a sustained, stable transduction. Ovine lentivirus vectors have the ability to infect a wide range of cell types from different species with similar efficiency to the well characterised murine leukaemia virus (MLV) vector. Self-inactivating vectors with non-active LTRs retain the ability to express transgenes *in vitro*. Integration deficient vectors appear to have to correct phenotype as eGFP-positive cells decreased over time in dividing cells.

## Conclusion

We have created a novel, self-inactivating, integration deficient ovine lentiviral vector which retains the ability to express transgenes *in vitro*. Future studies will investigate ovine lentiviral vectors mechanisms of immune response *in vitro* and study their efficiency to deliver appropriate immunity *in vivo*.

## References

[B1] Coutant F David RYS A nonintegrative lentiviral vector-based vaccine provides long-term sterile protection against Malaria PLoS One 2012 7 e48644 10.1371/journal.pone.0048644 23133649 PMC3487763

[B2] Zufferey R Dull T Self-inactivating lentivirus vector for safe and efficient in vivo gene delivery J. Virol 1998 72 9873 9880 9811723 10.1128/jvi.72.12.9873-9880.1998 PMC110499

[B3] Gaur M Leavitt AD Mutations in the human immunodeficiency virus type 1 integrase D,D(35)E motif do not eliminate provirus formation J. Virol 1998 72 4678 4685 9573231 10.1128/jvi.72.6.4678-4685.1998 PMC109991

